# Handwritten signatures in neurological and psychiatric disorders: a systematic review with clinical and forensic implications

**DOI:** 10.1007/s10072-026-09266-z

**Published:** 2026-07-28

**Authors:** Sara Vitale, Giacomo Guidali, Stefano Zago, Nadia Bolognini

**Affiliations:** 1 Department of Psychology, University of Milano-Bicocca, Piazza dell’Ateneo Nuovo 1, Milan, Italy; 2 Neurology Unit, Foundation IRCCS Ca’ Granda Hospital Maggiore Policlinico, Milan, Italy; 3 Laboratory of Neuropsychology, Department of Neurorehabilitation Sciences, IRCCS Istituto Auxologico Italiano, Milan, Italy

**Keywords:** Signature, Handwriting, Neurological, Psychiatric, Dementia

## Abstract

**Background:**

Signature production represents a complex motor and cognitive behavior, highly automatized and repeated countless times throughout life. It ranks among the most durable graphic forms, often preserved even when writing processes are impaired. Studies examining signature characteristics in clinical cohorts are limited. The available evidence raises questions about its potential as a behavioral marker of neurological and psychiatric disorders.

**Methods:**

We conducted a PRISMA-compliant systematic review up to 4th December 2025 to summarize evidence on how neurological and psychiatric conditions may affect signature features. Eligible articles on this topic were searched in Scopus, Web of Science, and PubMed databases. Sixteen papers were included, and their results were systematically described and qualitatively analyzed.

**Results:**

The available literature suggests that signature production appears relatively independent of global cognitive decline, although it is sensitive to specific pathological conditions (e.g., micrographia in Parkinson’s disease, increased tortuosity in cerebral small vessel disease, and alterations observed in mania, delirium, obsessive-compulsive disorder, and major depressive disorder). Alterations commonly affect kinematic parameters, as well as visuospatial and constructional features. However, no disorder-specific signature pattern emerges from current evidence, largely due to the methodological heterogeneity across studies.

**Conclusions:**

The handwritten signature holds promise as a behavioral marker of brain disorders, serving as a potentially useful, complementary - rather than a stand-alone - indicator of neurological and psychiatric conditions, which could be potentially useful in clinical settings. The implications for forensic applications remain largely theoretical and have not yet been standardized.

## Introduction

Handwriting represents the expression of a complex motor skill crucial in our daily lives, as it is a universally recognized method for authenticating documents and verifying people’s identities. Its execution relies on a widespread cerebral network that coordinates linguistic, perceptual, visuospatial, and praxic processes, making handwriting particularly sensitive to brain damage [1]. For this reason, in recent years, alterations in this ability as marker of cognitive functioning or even as pre-clinical/prodromic symptom of neurodegeneration have been investigated in various neurological and psychiatric populations, including Alzheimer’s disease (AD) [2–9], Parkinson’s disease (PD) [10, 11], Huntington’s disease [6, 12], dementia with Lewy bodies [13], bipolar disorder [14], schizophrenia [15], or obsessive-compulsive disorder (OCD) [16].

From a methodological standpoint, handwriting in clinical populations has been investigated through either offline (post-writing) or online (during-writing) analysis. Offline analysis focuses on the static, final written product: it examines the written product post-execution, typically visually assessing features such as text length (i.e., number of words), legibility, types of errors (e.g., orthographic, morphologic, allographic, omissions, perseverations), and spatial organization [17, 18]. In contrast, online analysis monitors the handwriting process in real time (e.g., speed, pressure, execution time) by tracking and recording the graphomotor trace to extract kinematic and spatiotemporal data, including speed, pressure, and execution time [19]. Overall, through these two approaches, it has been demonstrated that handwriting analysis can provide consistent, objective evidence to support clinical observations, focusing on both the final product and the underlying writing process. Thus, handwriting is becoming a central element in the symptomatic profile of various neurological and psychiatric disorders [20–22].

Among handwritten behaviors, the signature is a highly overlearned graphic gesture, repeated countless times throughout an individual’s life, that can provide a wealth of information beyond the signer’s name and surname [23]. Indeed, the act of signing is a rapid, spontaneous, and highly automatized motor gesture that requires minimal conscious control during execution [8, 24]. Due to its repeated use throughout life, the signature ranks among the most durable graphic forms, often preserved even when writing processes are impaired [8, 18]. Signature consistency may not extend to the entire graphic trace, but rather to specific portions of it, i.e., the so-called stable regions, defined as the longest sequences of similar pen strokes present in signatures produced by the same individual. These regions appear to capture distinctive features that are relatively resistant to writing impairment [24]. From a neurophysiological perspective, automated movements such as signing rely on a distributed neural network involving the parietal cortex, motor cortex, basal ganglia, and cerebellum. In particular, Marcelli et al. (2013) suggest that signature is represented as a motor plan distributed across subcortical structures, including the basal ganglia and cerebellum - specifically the dentate nucleus. These subcortical structures play a crucial role in the speed and agility of signature execution [25].

Traditionally, research on signatures has focused on personal biometric authentication [26, 27] or on a graphological perspective, examining the relationship between signature and personality traits [28, 29]. Systematic and objective investigations in clinical contexts, however, remain scarce, since handwriting research mainly focuses on writing words, phrases, and so on, by dictation or copy, or drawing. Studies examining signature characteristics in both healthy and clinical populations are limited and fragmented, often focusing on different measures and methodologies [8, 30–32]. Such heterogeneity largely reflects differences in how signatures are conceptualized and operationalized across recording modalities. Some studies analyzed signatures employing ‘physical’ traces (i.e., pen-and-paper or digitized versions of the ‘whole’ handwritten traces). Notably, this approach cannot provide information on the signing process, as the signature is treated as an ‘all-or-nothing’ variable. Conversely, studies that record spatiotemporal parameters online during signature execution enable the description of the temporal and kinematic properties of the writing process [33]. This latter approach allows for focusing on the dynamic parameters that characterize the act of signing, such as pen stroke fluidity, speed regularity, and pen pressure homogeneity [34], in turn identifying specific graphomotor parameters that may or may not be indicative of altered conditions and that might not be visible in post-writing (offline) analysis.

In recent years, experimental studies have begun to investigate whether and how the handwritten signature is affected by neurological and psychiatric diseases, particularly neurodegenerative diseases [8, 18]. These findings hold significant implications for both clinical and forensic practice. In clinical and legal contexts, the signature, far from being a mere motor act, reflects an individual’s ability to understand and approve the content of a document. This issue is particularly relevant in patients with brain disorders, in whom cognitive deficits can impair decision-making abilities. In the context of informed consent, a valid decision requires the ability to understand relevant information, evaluate its implications, reason about available options, and communicate a choice [35]. Following a stroke, for instance, one or more of these abilities may be impaired (e.g., comprehension may be impaired in patients with receptive aphasia; awareness of one’s own condition may be reduced in the presence of anosognosia; the expression of a coherent choice may be impaired by expressive aphasia). Nevertheless, the ability to provide a valid signature is rarely formally assessed in clinical settings. This represents an important gap in clinical practice, with implications extending beyond healthcare decision-making to the medico-legal domain, where both the authorship of a signature and the signer’s decision-making capacity at the time of signing may subsequently be called into question. Documents such as wills, deeds, and contracts containing disputed signatures are often submitted to forensic document examiners for analysis [30], and recently it has been proposed that retrospective evaluations regarding an individual’s cognitive and mental status may also take into consideration handwriting, particularly within the context of testamentary and contractual capacity assessments [36, 37]. To date, the results of these studies have not been systematically reviewed, which could provide an overview of consistent or contradictory evidence.

Given the legal and alleged clinical and diagnostic implications, in this work we reviewed studies that have explored pathological alterations in handwritten signatures, operationally defined as personalized habitual graphic traces produced by a person, primarily serving as a means of identity verification or legal endorsement. The broad spectrum of clinical conditions studied to date involves heterogeneous pathophysiological mechanisms that can alter signature production at multiple levels, ranging from sensorimotor abnormalities to single- or multi-domain cognitive deficits. In particular, the conditions in which the signature has been studied include neurodegenerative diseases (e.g., AD, frontotemporal dementias), characterized by progressive brain atrophy; vascular conditions (e.g., stroke), which compromise brain networks though focal or multifocal acquired lesions; acute encephalopathies (e.g., delirium), which involve transient alterations in central nervous system function and metabolic status; psychiatric disorders (e.g., OCD, major depressive disorder), which primarily involve neurotransmitter dysfunctions along with an aberrant functional connectivity, without macroscopic structural damages.

Our review aims to provide an overview of the current state of the art on signature handwriting across different neurological and psychiatric conditions, examining whether these conditions may uniquely influence specific signature characteristics. By integrating findings from studies conducted using both offline and online paradigms, we investigate the potential of handwritten signatures as an objective, non-invasive behavioral marker for early diagnosis and disease monitoring, while also exploring its critical implications for forensic assessment, addressing methodological limitations and outlining future directions for the intersection of clinical and forensic research in the field of handwriting.

## Methods

We conducted our systematic review in accordance with the evidence-based criteria outlined in the Preferred Reporting Items for Systematic Reviews and Meta-Analyses (PRISMA 2020) guidelines.

We systematically searched eligible articles on handwritten signatures in neurological and psychiatric diseases in the PubMed, Web of Science, and Scopus databases. The following search string was applied: ‘((signature) OR (signing) OR (kinematics) OR (graphomotor)) AND ((handwriting OR dysgraphia)) AND ((neurodegenerative OR stroke OR psychiatric OR cognitive impairment)). No filters regarding publication location or participants’ age group were selected during the search process. The literature search was conducted on 4th December 2025. We registered the present review on PROSPERO (International Prospective Register of Systematic Reviews – ID: CRD420261285396).

Two researchers (S.V. and G.G.), blinded to each other’s choices, independently extracted and selected articles, using Rayyan, an online platform for systematic reviews [38]. Disagreements regarding a study’s eligibility were resolved by consensus after article screening and selection. Across the three databases, the search yielded 1007 papers, which were reduced to 681 after duplicates were removed. We subsequently excluded studies that did not fit the aim of the present review. Specifically, we excluded studies if they met at least one of the following criteria:

- studies conducted only on a healthy cohort;
- studies investigating pathological handwriting in a clinical cohort, but without investigating signature ability;
- studies focusing on online signature verification/authentication;
- methodological papers implementing signature analysis using machine learning models, convolutional neural networks, or deep learning approaches.
- studies investigating signature characteristics from a graphological perspective (e.g., focusing on the relationship between signature and personality traits);
- reviews, meta-analyses, or conference abstracts;
- studies not published in peer-reviewed journals.

**Fig. 1:**
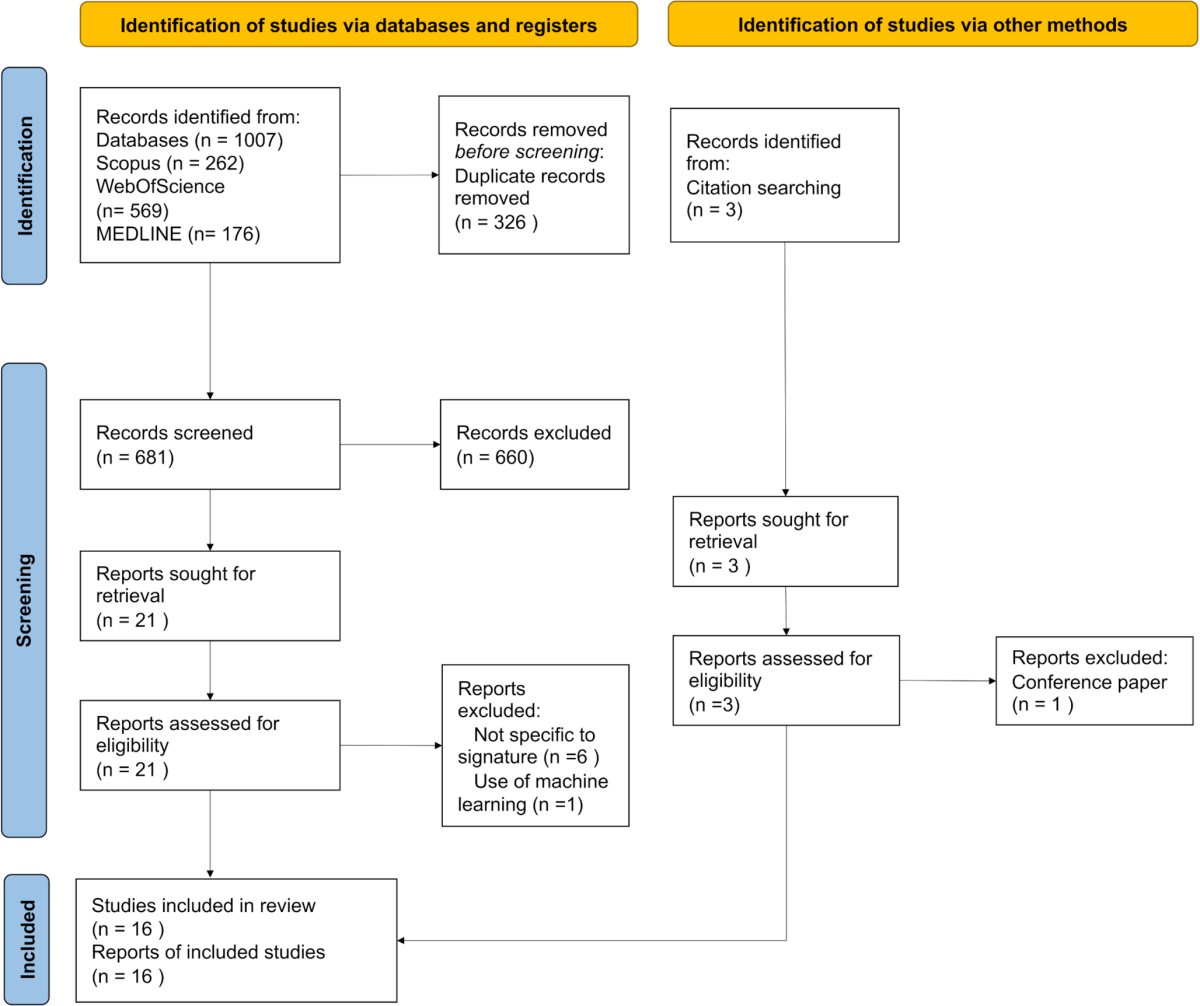
PRISMA flowchart illustrating the literature search and selection process for the present systematic review

After abstract screening, the blinding was removed. Eleven of 24 articles revealed incongruent evaluations among judges, which were discussed and resolved through consensus after a full-text eligibility assessment. After this step, we excluded 8 articles. Hence, a total of 16 papers (14 studies on a clinical population and 2 single-case reports) met the inclusion criteria and were included in the qualitative analysis. The PRISMA flowchart is shown in Fig. 1.

For each included study, the following data were extracted and systematically recorded by two reviewers (S.V. and G.G.): authors and year of publication; sample demographics; clinical characteristics and study design. Table 1 summarizes the extracted data. Due to methodological heterogeneity (see Table 1), statistical analyses of the extracted data could not be conducted; however, the main quantitative patterns are reported in Tables 3, 4, 5, 6, and 7 for completeness.

**Table 1:** Summary of extracted data

Study type	Study	Sample		Etiology	Experimental design	Evaluation type	Signature sample	Data source
		Patients	Healthy controls					
Population study	Baig et al., 1984	Schizophrenia = 45 Depression = 49 Mania = 15 Mental Disability = 11 Organic mental disorder = 50	*N* = 100	Psychiatric	Retrospective	Offline	Static	Archives (from patients’ medical records)
	Mavrogiorguo et al., 2001	OCD = 22 Age = 33.6(11.8)y	*N* = 22 Age = 34.9(13.5)y	Psychiatric	Cross-sectional	Online	Dynamic	Experimental Task (Wacom Tablet + Magnetic ball point Pen)
	Adamis et al., 2006	Medical Inpatients = 72 Age = 83.1(6.3)y	/	Neurological	Retrospective	Offline	Static	Archives (from consent form)
	Rosenblum et al., 2010	MDD = 20 Age = 75.7(6.61)y Education = 12.25(2.69)y	*N* = 20 Age = 74.55(7.63)y Education = 13.60(3.57)y	Psychiatric	Cross-sectional	Online	Dynamic	Experimental Task (Wacom Tablet + Inking Pen)
	Whitelock et al., 2015	Drug-dependent = 50 Age (range) = 18-55y	Siblings = 50 Unrelated HC = 50 Age (range) = 18-55y	Psychiatric	Retrospective	Offline	Static	Archives (from consent form)
	Pirlo et al., 2015	AD = 30	*N* = 32	Neurological	Retrospective	Online	Dynamic	Archives (from digital device)
	Renier et al., 2016	MCI = 36 Dementia = 38 Age (range) = 67-89y Education(range) = 2-17y	/	Neurological	Retrospecitve + Cross-sectional	Offline	Static	Archives (from identity card) + Experimental Task (paper-and-pencil)
	Zhi et al., 2017	PD = 10 Age (range) = 55.3-74.8y	*N* = 10	Neurological	Cross-sectional	Offline	Static	Experimental Task (paper-and-pencil)
	Fernandes & Lopes Lima, 2017	AD = 17 Age=80.88(4.26)y Education = 6.53 (3.02)y Severe AD = 17 Age = 79.53(8.37)y Education = 6.12 (3.43)y	*N* = 30 Age =78.6 (6.34)y Education = 6.47 (2.96)y	Neurological	Cross-sectional	Offline	Static	Experimental Task (paper-and-pencil)
	Israely & Carmeli, 2017	Post-stroke = 18 Age = 76.11(7.19)y	*N* = 19 Age = 71(10.58)y	Neurological	Cross-sectional	Online	Dynamic	Experimental Task (Computerized Penmanship Evaluation Tool)
	Wang et al., 2019	Early AD = 31 Age = 81.5 (7.4)y	*N* = 39 Age = 75.5 (6.4) y	Neurological	Cross-sectional	Online	Dynamic	Experimental Task (Wacom Tablet + Inking Pen)
	Caligiuri & Mohammed, 2019	AD = 69 Age = 75.56 (9.44)y	*N* = 74 Age = 74.92 (7.65)y	Neurological	Cross-sectional + Longitudinal	Online	Dynamic	Experimental Task (Wacom Tablet + Non Inking Pen)
	Preti et al., 2023	AD = 54 Age = 73.8 (6.4)y Education = 9.3 (4.3)y FTD = 49 Age = 70.4 (5.8)y Education = 10.1 (4.6)y	*N* = 31 Age = 73.1 (10.7)y Education = 10.6 (4.7)y	Neurological	Retrospective	Offline	Static	Archives
	Zhao et al., 2024	Severe CSVD = 16 Age = 71.25 (5.77)y Education = 9.69 (4)y Non-severe CSVD= 12 Age = 69 (8.60)y Education= 12.67 (3)y	*N*=32 Age = 65.66 (4.75)y Education = 11.88 (2.80)y	Neurological	Cross-sectional	Online	Dynamic	Experimental Task (Wacom Tablet + Non Inking Pen)
Case Study	Garcia, 2010	52y, F	/	Neurological	Single-case report + Longitudinal (from 52y to 91y)	/	/	/
	Bilieri et al., 2021	55y, M 61y, M	/	Neurological	Case reports	/	/	/

For each study described in the present review, the table reports: study type; authors and year of publication; sample size (divided for patients and healthy controls, specifying number of subjects, age [mean (SD) in years] and education [mean (SD) in years] if available; experimental design; evaluation type; signature sample; data source. *OCD,* obsessive-compulsive disorder; *MDD,* Major depressive disorder; *AD,* Alzheimer’s Disease; *MCI,* Mild Cognitive Impairment; *PD,* Parkinson’s Disease; *FTD,* Fronto-temporal Dementia; *CSVD,* Cerebral Small Vessel Disease

The internal validity of the selected articles was assessed using the Joanna Briggs Institute (JBI) Critical Appraisal Checklists for cross-sectional studies and case reports, according to each study’s design (retrieved from: https://jbi.global/critical-appraisal-tools). These tools are intended to evaluate the extent to which potential sources of bias have been addressed in the study design, conduct, and analysis [39–41]. Each checklist item was rated as ‘yes’, ‘no’, ‘unclear’, or ‘not applicable’ (Table 2). Following criteria established in previous reviews [e.g., 42, 43], the overall methodological quality was categorized based on the percentage of ‘yes’ responses as low (< 33%), medium (34%- 66%), or high quality (≥ 67%), corresponding to low, moderate, and high risk of bias, respectively. The assessment was conducted independently by two researchers (S.V. and G.G.), with any disagreements resolved by consensus.

**Table 2:** Risk of bias evaluation of included articles based on JBI critical evaluation tools

Cross-sectional studies										
Study	Q1	Q2	Q3	Q4	Q5	Q6	Q7	Q8	‘Yes’ percentage	Study quality
Adamis et al., 2006	Y	Y	Y	Y	N	N	Y	Y	75%	High
Baig et al., 1984	Y	Y	Y	Y	N	N	Y	Y	75%	High
Caligiuri & Mohammed, 2019	Y	Y	Y	Y	U	Y	Y	Y	88%	High
Fernandes & Lopes Lima, 2017	Y	Y	Y	Y	Y	Y	Y	Y	100%	High
Israely & Carmeli, 2017	Y	Y	Y	Y	Y	Y	Y	Y	100%	High
Mavrogiorgou et al., 2001	Y	Y	Y	Y	Y	Y	Y	Y	100%	High
Pirlo et al., 2015	U	U	Y	Y	N	N	Y	Y	50%	Moderate
Preti et al., 2023	Y	Y	Y	Y	Y	Y	Y	Y	100%	High
Renier et al., 2016	Y	Y	Y	U	N	N	Y	Y	63%	Moderate
Rosenblum et al., 2010	Y	Y	Y	Y	Y	Y	Y	Y	100%	High
Wang et al., 2019	Y	Y	Y	Y	Y	Y	Y	Y	100%	High
Whitelock et al., 2015	Y	Y	Y	Y	Y	Y	Y	Y	100%	High
Zhao et al., 2024	Y	Y	Y	Y	Y	Y	Y	Y	100%	High
Zhi et al., 2017	U	U	Y	Y	U	U	Y	Y	50%	Moderate
*Case Report*										
Study	Q1	Q2	Q3	Q4	Q5	Q6	Q7	Q8	‘Yes’ percentage	Study quality
Bilieri et al., 2021	Y	Y	Y	Y	N/A	N/A	N/A	Y	100%	High
Garcia, 2010	U	U	N/A	N	N/A	N/A	N/A	Y	25%	Low

*Y,* yes; *N,* no; *U,* unclear; *N/A,* not applicable

## Results

This review included sixteen articles that met our inclusion criteria, grouped into two main sections: studies investigating handwriting signatures in neurological disorders (*n* = 12; Tables 3, 4, 5, and 6) and studies focusing on psychiatric disorders (*n* = 4; Table 7). Depending on the study design, signatures were collected either prospectively during experimental or clinical activities or retrospectively from existing documents (e.g., identification documents, consent forms, or archives). We classified signatures as ‘static’ when analyses focused exclusively on the final written product, whether as an ink trace produced on paper or as a digitized version of a handwritten signature containing only graphical information. Conversely, signatures were classified as ‘dynamic’ when digitizing devices were used to capture online spatiotemporal and kinematic parameters during the signing process. Specifically, five studies retrospectively analyzed static signature samples obtained from medical records, consent forms, or identity cards. Two studies collected static signatures during experimental sessions using a paper-and-pencil paradigm. Only one study compared two static signature samples: one produced at the time of the evaluation and the other executed in the past (from the identity card). Six studies acquired dynamic signature samples using digitizing tablets. Two studies were single-case reports. For prospective studies, all except one [44; see 3.2] stated that participants had to make their own, genuine signature during the experimental task in which the signature is collected.

**Table 3:** Handwritten signature characteristics evaluated in AD

Study	Pathological condition	Signature sample	Parameters explored	Neuropsychological measures	Findings	Statistically significant quantitative patterns
Garcia, 2010	AD	Signatures executed over a period of 40y	Spatial impairments; dyskinesias; morphological alterations; omissions, additions, perseverations, substitutions of letters; bradygraphia, macrographia, lack of rhythm.	/	• Signature is a poor indicator of deterioration in the initial and intermediate dementia’s stages and becomes informative mainly in the final stages. • Reinforced learning makes the first name more resistant to graphic degradation than surnames. • Three stages of signature deterioration are described: dyssynergic, dyskinetic, and graphic drawing phase.	N/A
Pirlo et al., 2015	AD	From database	Command amplitude; neuromuscular system delays; response times; maximum speed; number of lognormal curves; number of peaks in the speed/time graph.	/	• Handwritten signature analysis based on the Sigma–Lognormal model may allow prediction of neurodegenerative disorders, supporting early AD diagnosis through kinematic key-features and a bagging CART classifier.	N/A
Renier et al., 2016	MCI, AD, VaD	Experimental task: two signature samples - one produced at the moment and one from identity card; spontaneous text.	Index of deterioration of signature: values between 0 = all evaluators agree on ‘‘very similar signatures’’ and 10 = all evaluators agree on ‘‘dissimilar signatures’’.	MMSE, ENPA, prose memory, forward and backward digit span, attentional matrices, TMT A and B, copy of drawings, CDT, FAB Stroop test, CET Tower of London, phonemic fluency task (F.A.S. version).	• No correlations were found between signing ability and spontaneous writing or cognitive functioning (MMSE), indicating that signature graphic integrity, due to its automaticity, is independent of cognitive status and represents an unreliable indicator of pathological cognitive decline.	N/A
Fernandes & Lopes Lima, 2017	AD	Experimental task: Signing for 10 times	General features: legibility, tremor and line quality, velocity, pressure, slant, curvature, overall size, spacing between words. Constructional features: perseverations, substitutions, omissions of strokes and letters, additional writing.	MMSE	• AD signatures show less legibility, tremor, poorer line quality, irregular word spacing, variable and irregular baseline, and constructional errors such as repetitions, omissions, substitutions, and unrelated additions. • Features like velocity, pressure, slant, curvature, and overall size showed no significant differences.	Prevalence of tremor: • Mild AD = 23.5% • Severe AD = 64.7% • HC = 16.7%
Wang et al., 2019	AD	Experimental task: Signing a check	Entropy measures calculated on raw temporal functions: pen position, pen pressure, pen inclination angles: azimuth and altitude.	MMSE	• Online signatures written by early-stage AD patients show significantly lower entropy values (lower irregularity and complexity), than those of healthy controls	Support vector machine classifier • Accuracy = 75.71% • Specificity = 76.92% • Sensitivity = 74.19%
Caligiuri & Mohammed, 2019	AD	Experimental tasks: signing for five times (re-test after 1y)	Stroke duration (in seconds), absolute vertical stroke amplitude (in cm), peak vertical stroke velocity (in cm/s), average normalized jerk, pen pressure.	DRS; UPDRS	• Dynamic signature features for AD subjects did not differ from controls and remained stable over one year (despite cognitive decline). • Greater dementia severity increased variability in stroke amplitude and speed for non-text-based signature, while text-based signatures were largely preserved	Non-text-based signature: AD = 14.5% vs. HC = 2.7%; Dynamic features variability: AD sample: ranged from 12.8% (for temporal and spatial features) to 43.2% (for fluency); HC samples: 10%
Preti et al., 2023	AD, FTD	Two signatures for each patient: one from the identity card (pre-diagnostic period), and one from the informed-consent document for CSF examination (after the diagnosis)	Signatures change score: 0 (= very similar signatures) or 1 (= different signatures); based on signature features: spatial layout, omission and/or addition and/or exchange of letters; omission and/or addition and/or exchange of names; changes of the shape of letters; changes of the pen-flow.	MMSE	• Signing ability is independent from cognition, age, and education in AD and FTD (no significant correlations between the ‘signature change score’ and the clinical and demographic factors were found). • Signature features that appear to observers to change the most were the pen-flow and the letters shape.	Signature change score (M ± SD) • AD: 35.6% ± 32.07% vs. HC:16.9% ± 24.5% • FTD: 43.9% ± 34.4% vs. HC:16.9% ± 24.5% Signature features: • pen-flow (29%); • shape of the letter (29%); • spatial layout (10%), • omission, addition or exchange of letters (3%) or of names (16%)

For each study, the table reports: authors and year of publication; pathological condition; signature sample characteristics; handwriting parameters explored; neuropsychological assessments; main findings; and statistically significant quantitative patterns. Quantitative patterns include statistical outcomes and performance metrics reported by the authors. *AD,* Alzheimer’s Disease; *MCI,* Mild Cognitive Impairment; *VaD,* Vascular Dementia; *FTD,* Fronto-temporal Dementia; *HC,* healthy controls; *MMSE,* Mini-Mental State Examination; *ENPA,* Esame Neuropsicologico per l’Afasia; *FAB,* Frontal Assessment Battery; *CET,* Cognitive Estimation Task; *DRS,* Dementia Rating Scale; *UPDRS,* Unified Parkinson’s Disease Rating Scale; *N/A,* not available

**Table 4:** Handwritten signature characteristics evaluated in Parkinson’s Disease (PD)

Study	Pathological condition	Signature sample	Parameters explored	Neuropsychological measures	Findings	Statistically significant quantitative patterns
Zhi et al., 2017	PD	Experimental task: Handwritten static signatures of PD subjects vs. sample control signatures artificially reduced in size	Global Metrics: Area under HPP/VPP (Horizontal Projection Profile – Vertical Projection Profile); local Metric: Pixel Density Variation.	/	• Area under HPP/VPP differences display large fluctuations only in PD subjects, indicating micrographia-related alterations in ink distribution. • Pixel Density variation shows significant changes in PD samples, reflecting sensitivity to left-to-right shrinkage (progressive micrographia).	Signature changes – before vs. after diagnosis: • Signature area: 54.26%; • Ink deposit: 52.91% • Height: 36.79%; • Width: 35.43% • Horizontal/Vertical projection profile area: 52.29% PD group vs. artificially reduced signatures: • Pixel density variation change: +104.68% vs. +8.18%

For each study, the table reports: authors and year of publication; pathological condition; signature sample characteristics; handwriting parameters explored; neuropsychological assessments; main findings; and statistically significant quantitative indicators. Quantitative indicators include statistical outcomes and performance metrics reported by the authors. *N/A,* not available

**Table 5:** Handwritten signature characteristics evaluated in cerebrovascular disease

Study	Pathological condition	Signature sample	Parameters explored	Neuropsychological measures	Findings	Statistically significant quantitative patterns
Israely & Carmeli, 2017	Post-stroke	Experimental tasks: full name; signature; ID number; free short sentence; connecting dots	velocity per pen stroke (in cm/s); pressure on the paper during handwriting; time-in-air per pen stroke	/	• Post-stroke subjects showed significantly lower velocity per stroke during signing. • No statistically group differences were found in pressure and time-in-air.	Stroke group vs. healthy controls (M ± SD) • speed: 3.02 ± 1.75 vs. 6.46 ± 2.21 • pressure: 285.6 ± 178.08 vs. 380.22 ± 98.32 • in-air time: 2.4 ± 6.01 vs. 0.23 ± 0.97
Bilieri et al., 2021	Post-stroke	/	/	/	• Patients reported poorly legible signatures; lesions included small lacunar infarction of the left insula and external capsule (patient 1) and a small emorragic lesion in the posterior limb of the left internal capsule (patient 2).	N/A
Zhao et al., 2024	CSVD	Experimental tasks: signature; writing of Chinese characters (‘正’ and ‘永’); Archimedes’ spiral drawing.	absolute velocity; pen pressure; in-air length tortuosity	MMSE	• The severe CSVD group showed lower velocity and higher tortuosity during signature. • Tortuosity of the signature was remarkably higher in the severe CSVD group and associated with CSVD severity.	Severe CSVD vs. Non-severe CSVD vs. healthy controls (M ± SD): • average absolute velocity: 1.23 ± 0.34 vs. 1.82 ± 1.04 vs. 1.84 ± 0.72, *p* = 0.022 • tortuosity: 0.43 ± 0.22 vs. 0.28 ± 0.18 vs. 0.22 ± 0.17, *p* = 0.003

For each study, the table reports: authors and year of publication; pathological condition; signature sample characteristics; handwriting parameters explored; neuropsychological assessments; main findings; and statistically significant quantitative patterns. Quantitative patterns include statistical outcomes and performance metrics reported by the authors. *CSVD,* Cerebral Small Vessel Disease; *MMSE,* Mini-Mental State Examination; *N/A,* not available

**Table 6:** Handwritten signature characteristics evaluated in acute encephalopathy

Study	Pathological condition	Signature sample	Parameters explored	Neuropsychological measures	Findings	Statistically significant quantitative patterns
Adamis et al., 2006	Delirium	Signature from consent form + sentence from MMSE	Dichotomous classification into ‘normal’ or ‘abnormal’ based on the presence of significant disturbances in: spatial orientation; omission of words, illegibility, spelling	MMSE	• Abnormal signature was significantly more common in the group with cognitive impairment and delirium than in the group with cognitive impairment without delirium.	Prevalence abnormal signature: delirious patients= 54.5% vs. non-delirious patients = 18.2% vs. HC = 0%’

For each study, the table reports: authors and year of publication; pathological condition; signature sample characteristics; handwriting parameters explored; neuropsychological assessments; main findings; and statistically significant quantitative patterns. Quantitative patterns include statistical outcomes and performance metrics reported by the authors. *MMSE,* Mini-Mental State Examination; *HC,* healthy controls; *N/A,* not available

**Table 7:** Handwritten signature characteristics evaluated in psychiatric disorders

Study	Pathological condition	Signature sample	Parameters explored	Neuropsychological measures	Findings	Statistically significant quantitative patterns
Baig et al., 1984	Schizophrenia; Depression; Mania; Mental Disability; Organic mental disorder	From patients’ medical records (at hospital admission time)	Signature size (maximal length and width)	/	• Signature size in the Mania group is significantly larger than those of any other categories of psychiatric diagnoses. • Signature size of organic mental disorder is significantly larger than those of the normal group.	Signature size (M ± SD) • Mania group: 1358.40 ± 675.63 mm², • Organic disorders: 978.17 ±516.57 mm², • Schizophrenia: 889.13±630.99 mm², • Depression: 864.47±498.77 mm² • Mental disability: 801.27±409.25 mm² • HC: 722.15 ±320.19 mm²; Mania vs. all groups (*p*<0.05); Organic disorders vs. HC (*p*<0.05)
Mavrogiorguo et al., 2001	OCD	Experimental tasks: superimposed concentric circles; given sentence; signature; letter sequences	Number of changes of velocity direction per pen stroke; pen stroke peak velocity (mm/s); pen stroke length (mm); skewness coefficient (%): the ratio of the acceleration phase to the total movement time.	WST IQ score; BDI; HDRS; Y-BOCS	• Hand motor disturbances in OCD were task-dependent: patients showed lower peak velocity, shorter strokes and reduced acceleration signatures. • Age influenced writing but not signatures. • Significant correlations between severity symptom and the kinematics of signature were found (mean peak velocity and mean stroke length; skewness coefficient).	OCD group vs. HC (M) • Lower peak velocity: 77.82 mm/s vs. 112.5 mm/s; • Age at onset and velocity inconsistency: Pearson’s *r*=−0.46 • Y-BOCS and changes in velocity: Spearman’s *r*=0.52 • Y-BOCS and peak velocity / stroke length: Spearman’s *r*=0.46 • Y-BOCS and skewness: Spearman’s *r*=0.56
Rosenblum et al., 2010	MDD	Experimental task: write letters of the alphabet; writing one’s name; fill out a check; paragraph copying.	In-air time per stroke; stroke width; pressure.	MMSE; GDS	• MDD participants spent more time with pen in the air and applied significantly less pressure during writing one’s name. • Moderately significant correlations were found in all tasks between in-air time and pressure and the GDS score and between in-air time of three tasks (except writing one’s name) and the MMSE score.	MDD vs. healthy controls (mean ± DS) • In-air time: 0.33 ±0.14 vs. 0.21±0.11, *p*<0.05; • Stroke widht: 0.32 ± 0.12 vs. 0.42± 0.16, ns; • Pressure: 569.41 ± 88.37 vs. 666.65 ± 115.28, *p* <0.05 Classification accuracy: 84.2%
Whitelock et al., 2015	Drug-dependence	From informed consent form	Signing below/on the designated signature line	CANTAB; NART; BDI-II	• Drug-dependent participants were more likely to sign below the line than their unaffected siblings, who in turn was more likely to do so than unrelated controls. • Signature positioning may be a soft sign for impairment of mechanisms involved in visuospatial memory (significant main effects of signature type and group – were observed in visuospatial task but no on other cognitive tests).	Signature below the line: Drug dependers = 48% vs. Siblings = 28% vs. Control volonteers = 12%’

For each study, the table reports: authors and year of publication; pathological condition; signature sample characteristics; handwriting parameters explored; neuropsychological assessments; main findings; and statistically significant quantitative patterns. Quantitative patterns include statistical outcomes and performance metrics reported by the authors. *OCD,* obsessive-compulsive disorder; *MDD,* Major depressive disorder; *MMSE,* Mini-Mental State Examination, *CANTAB,* Cambridge Neuropsychological Test Automated Battery; *WST,* Wortschatztest; *HDRS,* Hamilton depression rating scale; *Y-BOCS,* Yale-Brown obsessive-compulsive scale; *NART,* National Adult Reading Test; GDS; *BDI,* Beck Depression Inventory

All studies assessed for risk of bias met the minimum standards for quality evaluation and were retained for this review. Overall, 12 studies met the criteria for high quality, 3 for moderate quality, and 1 case report for low quality. For cross-sectional studies, the primary sources of bias were related to confounding factors (i.e., insufficient identification of confounders and the lack of strategies to manage them; 5 studies out of 14) and sample allocation and selection (i.e., lack of clearly specified exclusion or insufficient details regarding sample characteristics;2 studies out of 14). The result of the methodological quality assessment is shown in Table 2.

Below, we summarize the main findings from all these studies.

### Handwritten signature in neurological conditions

#### Signature in neurodegenerative disorders

García (2010) investigated longitudinal alteration and preservation of handwritten signatures in an AD patient. Signatures obtained retrospectively and spanning 40 years (from ages 52 to 91) were visually analyzed. Signature analysis focused on graphic, spatial, motor, and morpho-syntactic handwriting features, including dyskinesias (e.g., interrupted pen strokes, tremor, micrographia), letter-form alterations, omissions, additions, perseverations, and substitutions. Signature deterioration emerged only in the advanced stages of the disease, with varying preservation across signature components: the first name showed greater resistance to graphic degradation than the surname. The author also proposed a 3-stage model of signature deterioration in AD: a dyssynergic phase (melokinetic apraxia) characterized by extreme slowness and imprecise movements; a dyskinetic phase (ideomotor apraxia) characterized by tremor and irregular pen strokes; a final graphic drawing phase, in which the signature has lost almost all recognizable writing features [25]. This theoretical framework is based on observational findings. Indeed, although motor impairment is not considered a core clinical feature of AD, changes in fine motor control and coordination during handwriting tasks may reflect secondary consequences of deficits in executive functions, visuospatial abilities, and motor planning associated with disease progression.

Subsequent research adopted computational methods to explore the potential of handwritten signatures as a biometric marker for early detection of neurodegenerative disorders, with a specific focus on AD [23]. By applying the Sigma–Lognormal model, which describes motor commands and temporal properties of the neuromuscular system underlying handwriting production, a signature can be seen as a vector summation of lognormal distributions [45]. The Authors analysed a dataset of 62 dynamic signatures from healthy participants and AD patients using different machine learning classifiers. Results showed that specific kinematic features – i.e., command amplitude, neuromuscular delays, response times, maximum speed relative to writing time, the number of lognormal curves composing the signature, and the number of peaks in the speed/time graph – can effectively discriminate between healthy and pathological conditions [23].

One study examined the relationships among signature ability, writing performance, and cognitive functioning [18]. In a sample of 36 individuals with mild cognitive impairment (MCI) and 38 participants with AD, vascular dementia, or mixed dementia, both current and retrospective signatures obtained from identity cards were collected. Additionally, a neuropsychological battery was administered to assess global cognitive functioning, executive functions, and writing abilities. Signature deterioration was quantified using a dissimilarity index between the current and previous signatures, with ratings from five independent evaluators. Results revealed no correlation between signature deterioration and cognitive impairment, as assessed by the Mini-Mental State Examination (MMSE) [46]. Furthermore, the deterioration index was unrelated to the temporal distance between the two signature samples and to spontaneous writing performance [18].

While Renier et al. (2016) addressed signature stability in relation to cognitive status, Fernandes & Lopes Lima (2017) focused on characterizing post-writing features associated with AD. Two categories of handwriting features were analysed: general features (i.e., legibility, tremor, line quality, letter connectivity, velocity, pressure, slant, curvature, overall size, spacing between words, baseline shape, and direction) and constructional features (i.e., letter formation, unusual letter designs, and errors such as perseverations, omissions, or substitutions). Participants were instructed to produce their usual signature 10 times with a pen. Signatures from AD patients showed reduced legibility, increased tremor, poorer line quality, irregular word spacing, and increased variability in baseline shape and direction. Constructional features were also affected, with frequent repetitions, omissions, and substitutions. In contrast, features such as slant, curvature, and overall size did not differ significantly between the AD and control groups. According to the authors, these features may have forensic relevance for evaluating disputed signatures in AD [47].

Two studies adopted an online approach to compare AD patient signatures with those of the healthy population [30, 48]. In these works, dynamic features were captured using a digitizing tablet. Specifically, Wang et al. (2019) compared the *sample entropy of the signature* between early-stage AD and healthy controls. This index, calculated from raw temporal functions such as pen position, pen pressure, and pen inclination angles (azimuth and altitude), quantifies irregularity in time sequences and has been related to individual signature style [e.g., 49]. Three experts visually labelled all signatures into (i) text-based signatures (all the allographs are legible), (ii) mixed (one or more but not all of the allographs are legible), and (iii) stylized (none of the allographs are legible) following previously reported criteria [50]. Results indicated that different signature styles did not influence entropy values. Moreover, online signatures from early-stage AD patients showed significantly lower entropy values, reflecting lower irregularity and complexity, compared to controls, particularly for pen coordinates, pressure, and altitude angle [48].

Caligiuri & Mohammed (2019) introduced a longitudinal design, retesting AD patients one year after baseline assessment. Different variables, including pen stroke duration, absolute vertical stroke amplitude, peak vertical stroke velocity, average normalized jerk, and pen pressure, were examined. As in [50], the Authors considered signature style using the same classification criteria. No significant differences in dynamic signature features were found between AD and age-matched healthy controls. Dementia severity was associated with pen stroke amplitude and speed variability only for non-text-based signatures, suggesting that cognitive impairments in AD may affect the execution of mixed or stylized signatures while sparing text-based signatures, which are more automatic. Furthermore, despite a significant decline in cognitive status over one year, dynamic signature features remained relatively stable in AD subjects [30]. The authors suggest that forensic document examiners should be aware that, in most individuals with AD, signatures remain largely unaffected by the disease; however, potential interactions between dementia severity and signature style may contribute to variability across repeated signatures.

Recently, another study [8] aimed to deepen understanding of the temporal evolution of signature features in patients with AD and frontotemporal dementia (FTD) by adopting a modified version of the rater-based approach developed by Renier et al. (2016). In this work, for each patient, two signatures were collected and retrospectively analyzed: one from an identity card dated to a pre-diagnostic period, and one from the informed-consent document for the clinical examination, obtained after the diagnosis. Global signature changes and specific features (i.e., spatial layout, omission/addition/exchange of letters and names, modifications of the shape of letters, and changes of the pen-flow) were compared between AD, FTD, and neurologically healthy individuals. Results showed that both patient cohorts (i.e., AD and FTD) exhibited a higher percentage of detected signature changes compared with controls, although these changes were described as minor. The signature features that appear to change the most were pen flow and letter shape. However, no significant correlation was observed between the signature change score and patients’ clinical and demographic variables, suggesting the signature may be an unreliable indicator of the cognitive status in AD and FTD [8].

Handwritten signatures have also been examined in other neurodegenerative conditions, such as PD [51], to capture micrographia, namely a decrease in the letter size of handwritten text [52]. Static handwritten signatures from healthy subjects and patients with symptomatic PD were collected and compared with artificially downsized control signature samples. Global metrics such as the signature’s global area (defined by the maximum vertical and horizontal pen’s stroke extents) or the area under the horizontal and vertical projection profile, were used to characterize consistent micrographia, while a local metric, the *pixel density variation*, was introduced to capture the left-to-right shrinkage characteristic of progressive micrographia. On the one hand, global metrics showed similar decreases in PD and in the artificially resized control signatures, indicating that overall size measures alone cannot reliably distinguish true micrographia. On the other hand, the area under the horizontal and vertical projection profiles showed greater fluctuations in PD signatures than in the control sample, demonstrating that micrographia alters ink distribution patterns. Finally, pixel density variation analysis revealed significant changes in PD signatures, confirming its sensitivity to left-to-right handwriting shrinkage and its ability to capture the progressive micrographia typical of PD [51].

#### Signature in cerebrovascular diseases

Compared to neurodegenerative disorders, fewer studies have investigated this topic in cerebrovascular conditions. A case report by Billeri et al. (2021) described two patients who complained of reduced signature legibility as an early symptom of stroke onset. One patient had a lacunar infarction involving the left insula and the left external capsule, while the other had a small haemorrhagic lesion affecting the posterior limb of the left internal capsule [53].

Beyond single cases, Israely and Carmeli (2017) systematically examined handwriting and signature performance in post-stroke populations using a computerized handwriting evaluation tool. Writing speed, pen pressure, and in-air time were analysed. Post-stroke subjects showed significantly lower velocity signing, whereas no significant differences were found in pressure and in-air time [32].

Recently, Zhao et al. (2024) used digitized handwriting analysis to investigate signature movement abnormalities in older adults with cerebral small vessel disease. Subjects were grouped according to the severity of white matter hyperintensities assessed using the Fazekas scale [54]: severe (Grade 3 – confluent lesions), non-severe (Grade 2 – early confluent lesions), or healthy (Grade 1 or 0 – punctuate or absence of lesions). Results showed that patients with severe cerebral small vessel disease exhibited lower velocity and higher tortuosity during the signature than non-severe patients and healthy controls, with signature tortuosity more strongly associated with disease severity [55].

#### Signature in acute encephalopathy

Through an offline analysis approach, Adamis et al. (2006) examined signature and handwriting characteristics in a sample of 72 participants, including 22 patients with delirium, a mental status phenotype due to acute metabolic encephalopathy [56], 33 patients with cognitive impairment without delirium, and 17 cognitively intact controls without delirium. Signatures were obtained from informed consent forms, while handwriting samples were collected from the spontaneous sentence writing of the MMSE. Signature performance was dichotomized as normal or abnormal based on the presence of significant spatial or motor disturbances in spatial orientation, omissions, illegibility, and spelling errors. Abnormal signatures were significantly more common in the group with cognitive impairment and delirium than in the group with cognitive impairment without delirium. In contrast, handwriting abnormalities were common in cognitively impaired patients, both with and without delirium [57].

### Handwritten signature in psychiatric disorders

The first study testing the hypothesis that patients’ signatures could be used as an additional clinical sign to support psychiatric diagnosis was conducted by Baig et al. in 1984. The Authors conducted a correlational study investigating the possible relationships between signature sizes and psychiatric diagnosis, i.e., schizophrenia, depression, mania, mental disability, or organic mental disorders. The signatures of 252 psychiatric patients were retrospectively derived from their medical records, and, after visual inspection, the maximal signature’s length and width were assessed. Signatures in the mania group were significantly larger than those of any other category of psychiatric diagnoses and the control group. In contrast, signatures of patients with organic mental disorders were considerably larger only when compared to the control group but not to the other psychiatric conditions [58].

Mavrogiorgou et al. (2001) investigated handwriting and drawing kinematics in 22 unmedicated patients with OCD and 22 healthy controls using a digitizing tablet. Kinematic parameters were analysed: number of changes in velocity direction per pen stroke; pen stroke peak velocity, pen stroke length, and skewness coefficient (e.g., the ratio of the acceleration phase to the total movement time). Patients with OCD showed significantly lower peak velocity, shorter pen stroke, reduced time spent in acceleration during sentence writing and signature production compared to controls. Age did not considerably influence signature kinematics. However, a higher age at disease onset was associated with an increased number of changes in velocity direction during letter sequences and reduced peak velocity inconsistency only during sentence writing. Furthermore, OCD symptom severity, as measured by the Yale–Brown Obsessive Compulsive Scale [59], was positively correlated with several signature kinematic parameters, including mean peak velocity, mean pen stroke length, and writing skewness coefficient [60].

Rosenblum et al. (2010) conducted a preliminary study on older adults with Major Depressive Disorder (MDD). Using a digitizing tablet, kinematics parameters of handwriting, such as in-air time per pen stroke, pen stroke width, and pressure, were extracted. Results highlighted differences between MDD and control participants on the pressure measure across all handwriting tasks, including one requiring participants to write their first and last names. However, the authors did not specify whether this task involved the participants’ habitual signatures or ‘standard’ name-writing execution. It is important to account for this ambiguity, since the graphomotor processes underlying a customary signature that is highly automated and those required for writing one’s name on demand may be partly distinct. Moderate correlations were found across all tasks between the Geriatric Depression Scale [61] score and in-air time and pressure, while MMSE scores showed associations with in-air time for three tasks, except writing one’s name. Furthermore, a discriminant analysis based on graphomotor parameters collected during this task showed strong capacity to discriminate between individuals with mild MDD and healthy controls, with an overall classification accuracy of 84.2% [44].

Finally, Whitelock et al. (2015) investigated the relationship between signature position (i.e., on or below a given line on which the signature had to be performed) and cognitive functioning in a clinic sample of drug-dependent patients recruited for a previous study by the same research group [62]. The Authors hypothesized that, in this cohort of patients, inappropriate signature positioning would be associated with a selective deficit in visuospatial abilities. To test this hypothesis, signatures were collected from consent forms, and participants then performed a comprehensive neuropsychological assessment, including the Cambridge Neuropsychological Test Automated Battery [63]. Results showed that individuals who signed below the line performed significantly worse on tasks assessing visuospatial learning and memory, but not on other cognitive tests. These findings led the authors to conclude that atypical signature placement may be a soft sign for impairment of mechanisms involved in visuospatial memory [64].

## Discussion

**Fig. 2:**
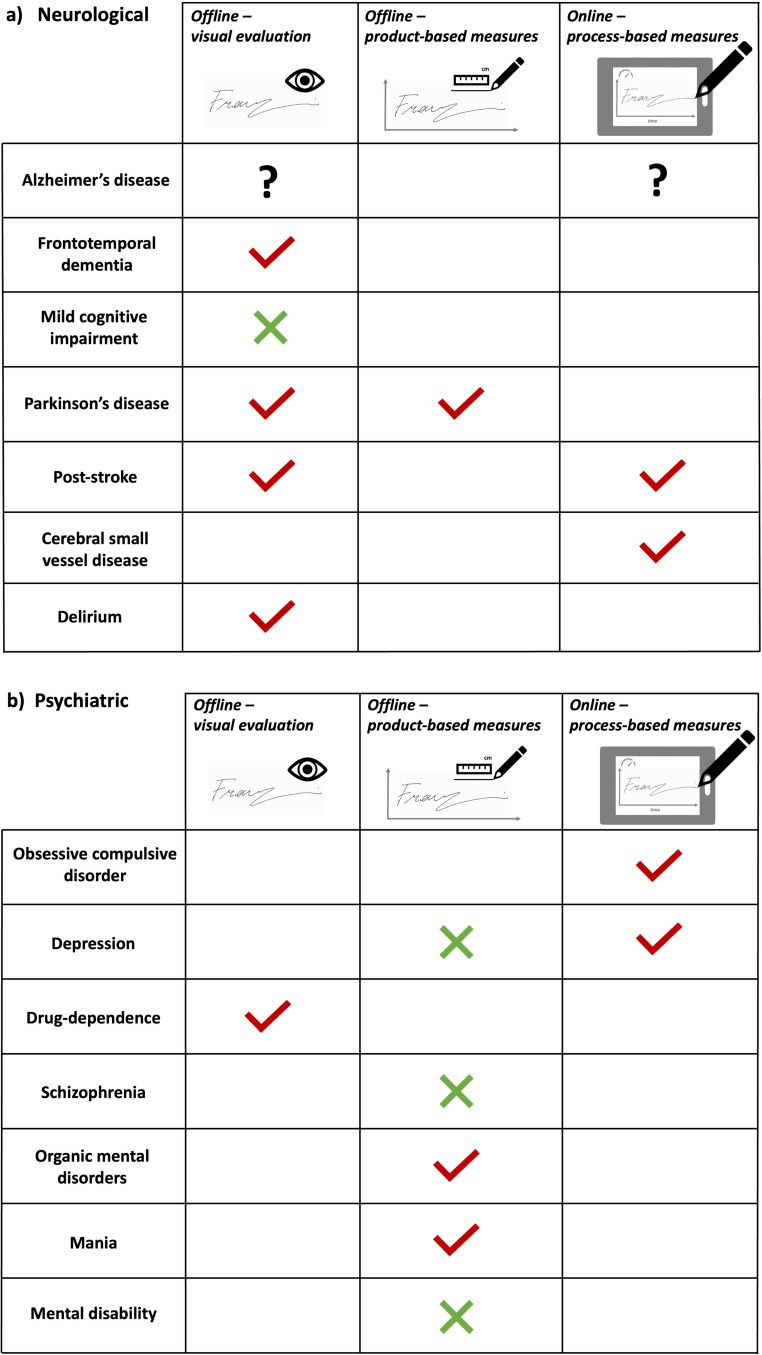
Evidence for post-writing (i.e., offline) and/or during-writing (i.e., online) alterations in signature production for neurologic (**a**) and psychiatric (**b**) disorders. Legend: ‘red thick’ = evidence of signature alterations; ‘green X’ = no evidence of signature alterations; ‘black question mark’ = inconsistency of evidence. See **Results** and **Discussion** sections for further information

Despite considerable methodological heterogeneity across studies, the literature reviewed here shows converging evidence that both offline and online analyses of patients’ signatures may be sensitive to different disorders affecting brain functioning, offering valuable directions and methodological recommendations for future research. A schematic representation summarizing whether offline (i.e., post-writing) or online (i.e., during-writing) signature alterations were found across neurological and psychiatric disorders is depicted in Fig. 2.

### Disorder-specific patterns of signature alteration

A first key finding is that kinematic parameters of signing, such as velocity, pen pressure, and in-air time, exhibit disorder-specific modulations. Reduced velocity is reported in cerebrovascular conditions, both in post-stroke patients and in individuals with severe cerebral small vessel disease, the latter also exhibiting increased in-air tortuosity between characters [32, 55]. In OCD, signature execution is characterized by lower peak velocity, shorter stroke length, and a reduced acceleration phase [60]. In contrast, major depressive disorder shows alterations in pressure and in-air time [44]. Notably, in the latter study, the authors did not explicitly clarify whether the task of ‘writing one’s name’ referred to producing a handwritten signature or was a simple name-writing task. This distinction carries crucial implications: while one’s own handwritten signature is an overlearned process that relies heavily on automated procedural memory and is often preserved even in severe cognitive decline, writing one’s name as a text-copying or generation task requires a higher degree of graphomotor control and cognitive processing. Therefore, these findings should be interpreted with caution, as they might reflect generalized psychomotor slowing rather than a specific degradation of the signature trace per se.

In AD, standard kinematic parameters such as velocity and pressure do not consistently differentiate patients from controls and may remain stable over time [8, 30, 47]. However, entropy-based measures derived from raw temporal signals reveal reduced motor complexity as early as the earliest AD stages [48], suggesting that subtle alterations may remain invisible when an offline visual inspection approach is employed. Alterations in visuospatial and global features can be observed in neurodegenerative and acute confusional states. In AD, reduced legibility, tremor, poorer line quality, irregular spacing, baseline variability, and constructional errors are described, and pen-flow and letter-shape modifications are most frequently perceived as changed, as observed in a longitudinal study [47]. It is worth noting that features such as slant, curvature, and overall size do not differ significantly between AD and controls, indicating that spatial variables are not equally sensitive to neurodegeneration [8]. Such discrepancies across studies in AD could be explained by evidence that motor impairment is not considered a core clinical feature of this neurodegenerative disease [65]. Consequently, alterations in fine motor control and coordination during signature tasks may represent secondary consequences of higher-order deficits (e.g., in executive functions or visuospatial abilities), thereby complicating the identification of common modulation patterns across the reviewed studies.

Similarly, in PD, global size alone does not distinguish micrographia; instead, projection-profile and pixel-density metrics detect altered ink distribution and left-to-right shrinkage patterns that characterize both consistent and progressive micrographia in handwritten signatures [51]. However, in psychiatric diseases, visuospatial modulations follow a different pattern: enlarged signature dimensions are explicitly associated with mania, potentially reflecting psychomotor activation, whereas atypical spatial positioning has been linked to poorer visuospatial learning and memory in drug-dependent individuals [58, 64].

Finally, constructional errors (e.g., omissions, substitutions, additions, perseverations) are reported in neurological conditions. In AD, these are described as characteristic alterations, and spelling errors and omissions seem more common in handwritten signatures from patients with delirium than in those from cognitively impaired patients without delirium [47, 57].

### Methodological considerations: offline vs. online approaches

Overall, offline and online studies converge in demonstrating the value of the signature as a graphic product that can provide information to support clinical observations. Both approaches inevitably operate at different levels of complexity and are complementary in what they reveal. Post-writing, offline, analyses capture apparent and macroscopic alterations such as omitted letters, tremors, or unusual graphemic features (e.g., shape or formation). Although offline approaches offer greater ecological validity in signature collection, they are susceptible to greater contextual variability, such as the conditions under which signatures were produced [e.g., available writing space, writing position, pen type − 33]. Moreover, offline evaluation is strongly influenced by the clinician’s experience, thereby increasing the risk of rater bias. These aspects limit the interpretability of results. However, they provide access to large datasets through historical documents (e.g., consent forms, identity cards). Retrospective analysis of signatures collected during the pre-diagnostic period or before a cerebrovascular event allows comparisons before and after clinical diagnosis and potentially provides information on longitudinal changes [8].

Nevertheless, by focusing only on the static final written product (i.e., the visual features of the signature), all kinematic and spatiotemporal information available during execution is lost. Conversely, during-writing, online, approaches provide objective measures of temporal and kinematic aspects of signature execution, even if the resulting signature is less detailed and less continuous than a paper-based one [33]. Dynamic signatures, by recording the spatiotemporal profile of the handwritten trace, allow the detection of subtle motor and cognitive features that may already be impaired in early or intermediate stages of neurodegenerative or cerebrovascular disorders [23, 48, 55].

Handwritten signatures also exhibit a physiological intra-individual variability due to normal fluctuations in motor control, attention, and neuromuscular execution [21, 33]. By recording multiple repetitions within the same session, during-writing methods allow researchers to characterize this baseline variability and distinguish it from disorder-related alterations. Furthermore, the literature reports variability in writing performance across writing surfaces: writing on a digitizing tablet alters pen pressure, letter size, and speed compared to writing on paper, and individuals unfamiliar with digital devices may require multiple sessions to stabilize performance and produce ecologically valid signatures [66]. Online approaches are also limited by heterogeneity in variables and protocols adopted across studies, which complicates comparisons and reduces generalizability.

Overall, it seems that post-writing and during-writing approaches are somewhat complementary and both informative: post-writing, offline, analysis remains valuable for identifying structural alterations, while during-writing, online, methods can transform signature analysis into a non-invasive potential behavioral marker capable of detecting subtle graphomotor impairments masked by an apparently preserved signature, allowing more sophisticated analyses of signature changes over time and across diseases.

### Signature and cognition

Current evidence suggests that handwritten signatures exhibit a peculiar resistance to cognitive decline, highlighting a potential dissociation between automated graphomotor writing routines and cognitive functioning. However, very few studies have systematically examined the neuropsychological correlates of signing. The available evidence frequently reports a lack of significant association between signature degradation and global cognitive scores when derived from screening tests such as the MMSE, or from the Dementia Rating Scale, which evaluates the progression and severity of cognitive deterioration [8, 18, 30, 44]. Even when an exhaustive neuropsychological battery was administered, as in the study by Renier et al. (2016), cognitive deficits correlated only with impairments in spontaneous handwriting, not with signatures, at least when analyzed with an offline, although structured, method, as done so far.

However, these negative findings must be interpreted in light of severe methodological limitations in the current evidence, rather than as definitive evidence of the absence of neuropsychological correlations. First, global cognitive screening tools, such as the MMSE, have significant limitations when used for comprehensive cognitive evaluation. Its main drawback is that it is heavily biased toward memory and orientation, largely neglecting executive functions. This makes it notoriously unreliable for detecting phenotypes of cognitive decline in which memory is preserved. Furthermore, the test is influenced by factors such as low education level or advanced age, which can lead to false positives. In general, cognitive screening tests serve as an initial filter, but they cannot replace a detailed neuropsychological evaluation, which is more sensitive in detecting whether deficits in specific cognitive functions may affect a person’s handwritten signature. On the other hand, using more sophisticated online graphomotor analyses, or presenting the signature task within specific structured conditions, might be more useful for detecting subtle alterations in cognitive functioning. Kinematic variations, such as increased in-air trajectories or erratic pen pressure, could be more likely to be associated with executive control, motor planning, or visually guided motor monitoring. For example, signing below a designated line has been shown to be affected by visuospatial processing impairments in drug-dependent individuals [64].

In summary, the interpretation of current evidence regarding the lack of correlations between cognitive functioning and changes in handwriting is limited by two main factors: the widespread use of global cognitive screening tests and the scarcity of studies that combine sophisticated graphomotor analyses with domain-specific second-level neuropsychological batteries. Only by systematically mapping specific cognitive subdomains will it be possible to fully clarify the clinical, prognostic, and forensic implications of signature analysis and transform it into a potentially reliable indicator of early cognitive decline.

### Underexplored cross-task evaluation: signature production vs. spontaneous handwriting

A key methodological gap in the current literature concerns the limited comparison between signature execution and spontaneous writing. It is well known that spontaneous writing is far more sensitive to early cognitive deterioration and progressive motor and linguistic decline [8]. This is not surprising, considering that spontaneous writing required a higher linguistic, cognitive, and motor load than signing, which is indeed a more automatized and stereotypical form of handwriting with minimal linguistic computation. However, comparisons between these two handwriting types have been conducted only sporadically [18, 32, 55, 57, 60]. For example, Renier et al. (2016) report no significant correlations between signatures and spontaneous writing performance [18]. Mavrogiorgou et al. (2001) found that in OCD, age (at the onset of the disease) is not associated with signature parameters, but is associated with kinematic variations in sentence writing [60].

Despite these sporadic contributions, systematic within-subject comparisons between signatures and spontaneous handwriting remain lacking. More consistent evaluation could clarify whether signature alterations represent a distinct graphomotor sign with specific clinical sensitivity compared to conventional handwriting tasks and standard writing assessments.

### Temporal resilience of signature production

Across the literature, signing is described as one of the most durable graphic gestures, likely due to its automatized and overlearned nature. Converging evidence from both offline and online studies supports this hypothesis [18, 25, 30]. Not only does signature execution appear to be resilient to cognitive decline, as discussed earlier, but it also shows an association with disease progression, exhibiting noticeable changes only in its advanced stages. Indeed, signatures often correlate more strongly with the severity of specific neuropsychiatric symptoms. For example, in patients with cerebral small vessel disease, signature tortuosity is associated with the severity of white matter hyperintensities, and dementia severity correlates with greater variability in stroke width and speed only for non-textual signatures [30, 55]. In OCD, symptom severity is associated with peak velocity, stroke length, and skewness coefficient [60]. Similarly, in major depressive disorders, depression severity correlates with kinematic modulations - reduced pen pressure and increased in-air time [44]. Again, delirium has been specifically associated with abnormal signatures characterized by spatial disorientation, omissions, and illegibility [57]. Overall, the signature appears relatively stable over time, but its spatiotemporal features are disrupted when symptom severity increases or when tested under acute conditions. However, further longitudinal investigations are required to clarify how signatures evolve with disease progression and their potential clinical value.

### Neural substrates of signature execution

From a neurobiological perspective, the relative resilience of signature execution and the disorder-specific alterations may be understood in terms of the distributed neural networks that support automated movements. Signature production relies on a distributed network involving the parietal cortex, motor cortex, basal ganglia, and cerebellum [24]. This framework could explain why signature execution appears relatively preserved in the early stages of neurodegenerative conditions such as AD, where overlearned and procedural motor routines may remain intact [8, 25]. Conversely, subcortical vascular damage, as observed in cerebral small vessel disease, may disrupt white matter tracts and fronto-subcortical connectivity, leading to increased motor variability and altered signature dynamics [55]. Evidence from OCD shows task-specific alterations likely due to impaired modulation of complex motor sequences, possibly linked to basal ganglia dysfunction [60]. Although direct neuroimaging evidence of the neural underpinnings of signature is absent, clinical reports of stroke cases indicate impaired signature legibility following damage to localized brain regions like the left insula, left external capsule, and the posterior arm of the left internal capsule [53]. Future studies combining kinematic analysis with structural and functional neuroimaging would be essential to clarify the neural mechanisms underlying both the resilience and vulnerability of this highly automatized gesture.

### Sources of variability in signature analysis: style, demographic, and medication effects

Additional factors that may influence signature performance, such as demographic characteristics (age, education) and medication, are not always reported across studies. It is well-known that medication and substance abuse may have effects on handwriting movements [6, 10, 33, 67]. Among the reviewed studies, only two explicitly addressed this issue. On the one hand, Fernandes & Lopes Lima (2017) reported potential effects of antipsychotic treatment on trace quality in AD [47]; on the other hand, Zhi et al. (2017), based on their data, concluded that medication neither improves nor impairs subjects’ handwriting [51].

Furthermore, while aging effects on handwriting have been documented, with younger individuals typically writing faster and more fluently than older adults [33, 68], signature performance appears relatively independent of age or education [8, 60]. These results further suggest that signatures are less vulnerable than automated gestures to demographic characteristics, at least in clinical populations in which this association has been investigated so far (e.g., AD, frontotemporal dementia, OCD).

Finally, the personal style of the signature writing should be considered another source of variability in clinical studies. Among the few studies that considered this aspect, Caligiuri & Mohammed (2019) reported a significantly higher proportion of non-text-based signature writers (i.e., allographs that are partially or completely illegible) among AD patients than among healthy controls. Moreover, text-based signatures (i.e., all the allographs are legible) appear to be largely preserved even in the presence of AD. Indeed, dementia severity was associated with greater variability in pen stroke amplitude and velocity only in non-text-based signatures (i.e., mixed or stylized). This may be explained by the fact that the textual style is considered more automatic than stylized or mixed forms [30]. Despite kinematic differences, online analysis using entropy measures revealed that movement complexity is not influenced by signature style [48].

Altogether, further investigations are needed to clarify the influence of style, demographics, and medication on signature performance.

### Clinical and forensic implications

The findings of this systematic review have significant practical implications, suggesting that signature alterations must be interpreted with extreme caution in both clinical and forensic contexts.

From a forensic perspective, the formal, ‘aesthetic’ quality of a handwritten signature on a legal document, such as a testament or a contract, should not be regarded as direct evidence of competence. An apparently well-preserved signature can easily coexist with cognitive impairment, particularly during the early stages of neurodegenerative disorders. This is because signature production relies heavily on highly overlearned, automated procedural motor routines, distributed across subcortical structures, that may be less vulnerable to cognitive impairments. Forensic experts should therefore be aware that what appears “visually” unchanged does not necessarily imply that cognitive functions are intact. From this perspective, signature analysis using digital acquisition [used online, in the case of simultaneous assessment of writing ability, or offline, for example, for retrospective assessment of writing ability − 69] may provide more relevant information for capturing subtle motor and kinematic alterations that cannot be detected through retrospective graphological examination of the final written trace alone. Conversely, evident alterations in signature execution do not automatically indicate generalized cognitive decline, hence a lack of legal competence; instead, they may reflect motor deficits (e.g. the progressive micrographia in Parkinson’s disease), but also specific cognitive deficits (visuospatial, executive deficits) or transient, reversible, medical conditions such as acute confusional states, which may or may not be relevant depending on the legal capacity under evaluation.

These considerations are also relevant in clinical settings, particularly in evaluating informed consent. While signing a consent form is standard practice for documenting a patient’s medical decision, clinicians must recognize that a well-executed signature is not proof of intact decision-making ability, just as a dysgraphic signature is not an automatic indicator of legal incapacity.

With respect to clinical implications, at the current state of the art, disorder-specific signature profiles have not yet emerged for diagnostic purposes, largely due to the methodological heterogeneity across studies. Nevertheless, in clinical routine and forensic assessments, the transition from traditional, retrospective graphological analysis to online signature analysis using digital devices will represent a significant step forward; however, this requires research to identify and standardize reliable markers of pathological alterations in signatures and to determine their relationship to cognitive functioning. To maximize the clinical and forensic utility of signature analysis as a complementary tool for tracking graphomotor impairments, cognitive deficits, and disease progression, future research must prioritize the adoption of standardized administration protocols and digitally-acquired kinematic metrics.

## Conclusion

Although the reviewed studies suggest that handwritten signatures are susceptible to clinical alterations that affect both the final written trace features and the kinematic parameters involved in handwriting execution, the current literature does not support establishing standardized criteria, cut-off values, or diagnostic thresholds applicable directly in clinical or legal settings. For these reasons, signature analysis should be considered as a complementary source of information that may contribute to clinical and forensic reasoning only when integrated with neuropsychological, neurological, and contextual evidence, rather than as a standalone indicator of cognitive and mental status for clinical diagnostic purposes or for forensic assessments of specific legal capacities, such as testamentary capacity. Nevertheless, the review highlights the need for more standardized and systematic approaches to signature evaluation. Based on the results, several recommendations can be made to guide future applied research.

Firstly, signature collection protocols had to be standardized. Future research should establish rigorous protocols for the online signature acquisition and the offline signature evaluation. To adequately capture intra-individual variability and stability under standardized conditions, protocols should include multiple signature repetitions within controlled testing environments. Furthermore, when using digital devices, preserving ecological validity is fundamental: future protocols should employ acquisition devices that closely approximate natural writing conditions and include a structured familiarization phase prior to testing to minimize performance distortions due to device novelty.

Secondly, it is necessary to identify a core set of graphomotor parameters to ensure comparability across clinical and forensic settings. This core set should include kinematic and spatiotemporal metrics (such as total execution time, in-air time, average pen pressure, kinematic fluidity/jerk, number of strokes), ideally normalized by signature length or complexity to account for individual signature styles. Quantitative kinematic measures should be supplemented by visual assessments of signature features (e.g., spatial alignment, structural disorganization) and error patterns [e.g., 17, 70], as well as comprehensive neuropsychological evaluations.

Thirdly, longitudinal studies are needed to track how signature alterations evolve alongside clinical progression and cognitive decline. Of great interest, the investigation of the relationship between longitudinal signature changes and legally relevant milestones, such as the execution of wills, the signing of contracts, or the longitudinal tracking of capacity to provide informed consent in medical settings. Such studies would help clarify whether specific signature drops predict or correlate with the loss of specific legal capacities.

In conclusion, the study of signatures requires further investigation as a window into the complex cognitive and graphometric processes that characterize this peculiar human behavior, reflecting procedural memory closely linked to our personality and identity, with specific developmental trajectories. Unveiling and understanding alterations in signatures caused by neurological and psychiatric diseases may have clinical significance for diagnosis and prognosis [23, 48, 51], as well as implications for forensic settings [36]. The added value of signature analysis may lie not in providing definitive conclusions about diagnosis, capacity, or authenticity, but in offering objective behavioral markers that can complement clinical and forensic evaluations.

## Data Availability

Data sharing is not applicable to this article as no datasets were generated or analyzed during the current study.
